# Preliminary Genomic Characterization of Ten Hardwood Tree Species from Multiplexed Low Coverage Whole Genome Sequencing

**DOI:** 10.1371/journal.pone.0145031

**Published:** 2015-12-23

**Authors:** Margaret Staton, Teodora Best, Sudhir Khodwekar, Sandra Owusu, Tao Xu, Yi Xu, Tara Jennings, Richard Cronn, A. Kathiravetpilla Arumuganathan, Mark Coggeshall, Oliver Gailing, Haiying Liang, Jeanne Romero-Severson, Scott Schlarbaum, John E. Carlson

**Affiliations:** 1 Department of Entomology and Plant Pathology, University of Tennessee, Knoxville, TN, United States of America; 2 The Schatz Center for Tree Molecular Genetics, Pennsylvania State University, University Park, PA, United States of America; 3 School of Forest Resources and Environmental Science, Michigan Technological University, Houghton, MI, United States of America; 4 Department of Genetics and Biochemistry, Clemson University, Clemson, SC, United States of America; 5 Department of Botany and Plant Pathology, Oregon State University, Corvallis, OR, United States of America; 6 Flow Cytometry and Imaging Core Lab, Benaroya Research Institute at Virginia Mason, Seattle, WA, United States of America; 7 Department of Forestry, University of Missouri, Columbia, MO, United States of America; 8 Department of Biological Sciences, University of Notre Dame, Notre Dame, IN, United States of America; 9 Department of Forestry, Wildlife and Fisheries, University of Tennessee, Knoxville, TN, United States of America; Aristotle University of Thessaloniki, GREECE

## Abstract

Forest health issues are on the rise in the United States, resulting from introduction of alien pests and diseases, coupled with abiotic stresses related to climate change. Increasingly, forest scientists are finding genetic/genomic resources valuable in addressing forest health issues. For a set of ten ecologically and economically important native hardwood tree species representing a broad phylogenetic spectrum, we used low coverage whole genome sequencing from multiplex Illumina paired ends to economically profile their genomic content. For six species, the genome content was further analyzed by flow cytometry in order to determine the nuclear genome size. Sequencing yielded a depth of 0.8X to 7.5X, from which *in silico* analysis yielded preliminary estimates of gene and repetitive sequence content in the genome for each species. Thousands of genomic SSRs were identified, with a clear predisposition toward dinucleotide repeats and AT-rich repeat motifs. Flanking primers were designed for SSR loci for all ten species, ranging from 891 loci in sugar maple to 18,167 in redbay. In summary, we have demonstrated that useful preliminary genome information including repeat content, gene content and useful SSR markers can be obtained at low cost and time input from a single lane of Illumina multiplex sequence.

## Introduction

North American forests have suffered extensive tree mortality and ecosystem disruption due to the introduction and establishment of invasive foreign insects and microbes [1–4] as well as abiotic stress due to climate change [5–7]. In light of the increasing pressures on forests and tree plantings, there is a growing need for tools to inform tree management, conservation and improvement as well as large reforestation efforts. Genetic and genomic resources are widely recognized as valuable assets for these activities by illuminating the associations between genotype, phenotype and environment [8]. High throughput, low cost sequencing chemistries are providing unprecedented opportunities for genomic resource development for a wide array of non-model species. Through paired end multiplex sequencing, we generated low-depth shotgun genome sequences from ten native hardwood tree species from the eastern United States: black cherry (*Prunus serotina* Ehrh.), black walnut (*Juglans nigra* L.), blackgum (*Nyssa sylvatica* Marshall), green ash (*Fraxinus pennsylvanica* Marshall), honeylocust (*Gleditisia triacanthos* L.), redbay (*Persea borbonia* (L.) Spreng.), sugar maple (*Acer saccharum* Marshall), sweetgum (*Liquidambar styraciflua* L.), white ash (*Fraxinus americana* L.) and white oak (*Quercus alba* L.). The species chosen have few existing genomic resources and represent a phylogenetically wide range of plant families. Many of the species have high economic value, importance in forest ecosystem function and/or pressure from invasive pests or pathogens.

Low coverage of genome reads is a common technique for profiling the genomic content of a species and generating new genomic resources. Referred to as genome survey sequencing (GSS), it has been used in animal, plant and insect species. Prior to the advent of next generation sequencing, a 1.5X shotgun sequencing of the canine genome was used to identify canine orthologs to mouse and human genes and to detect common repetitive element types [9]. A 0.66X of the pig genome also provided thousands of new mammalian gene orthologs and demonstrated that the pig genome is more similar to human than to mouse [10]. With much reduced cost to generate sequence data, GSS has been successfully utilized with next generation sequencing in barley, where kmer-based analysis of sequence data spanning less than 10% of the genome revealed novel repetitive elements [11]. In scuttle fly, pyrosequencing of 10% of the genome identified gene homologs to other Dipterans and a survey of repeat elements [12]. For milkweed (*Asclepias* L.) 0.5X coverage yielded not just genes and repeat elements, but also a whole chloroplast genome and a partial mitochondrial genome [13]. Most recently, six fern species were assayed for genome content by low coverage sequencing (.4X to 2X) with a goal to identify the most promising species for whole genome reference sequenicng [14]. These studies indicate that next generation sequencing can be successfully used to investigate and generate new resources from the genomes of non-model species.

Low coverage sequencing is also commonly used to mine the genome for molecular markers that can be used to assess population structure, population genetic diversity, gene flow between populations, and selective genetic pressures [15–18]. Microsatellites, or simple sequence repeats (SSRs), are molecular markers common throughout plant genomes and often transferable across closely related species [19]. Initially hampered by short read lengths, the development of paired end sequencing and the increase in sequence lengths have opened the Illumina platform to inexpensive, high throughput SSR discovery. This has been successfully demonstrated with both microsatellite-enriched libraries [20,21] and unenriched genomic DNA [13,22]. Such large volumes of data are produced in a single lane of Illumina sequencing that multiple individuals and species may be surveyed simultaneously through multiplexing, i.e. barcoding and pooling sequence libraries together [21].

In addition to low coverage sequencing, we used flow cytometry to estimate nuclear DNA content for six of the ten sequenced trees. The genome size estimates make the genomic content information from the sequencing more valuable as the percentages of genic and repetitive content can be converted into estimates of base pairs. This understanding of genome complexity and size is important for future genomic resource planning and development. Certain applications that rely on depth of sampling across the physical genome, such as whole genome sequencing or genomic clone library construction, cannot be effectively undertaken without knowledge of genome size. Cytological results also inform plant breeding strategies [23], particularly intraspecific crosses [24], and can contribute information to phylogenetic relationships between taxa and species boundaries [25].

The genome sequence data and genome size information serve as a base for building further genomic and genetic experimentation including genetic and QTL (quantitative trait loci) map development, genetic association, genomic selection, and whole genome sequencing, all of which may be useful for elucidating the genetic basis of complex traits [26,27]. Genomic resources can be used as tools for the management and conservation of important tree species by identifying populations under pressure from climate change and estimating adaptive potential and genetic diversity of germplasm resources [28,29]. The vast majority of North American hardwood tree species do not have tree breeding programs, but for those that do, molecular markers can increase the efficiency and speed of genetic improvement [30,31]. In addition to tree improvement applications, these resources provide new information for comparison of plant genomes across large phylogenetic distances. Four of the trees represent taxonomic families without a reference genome or prior genome survey sequencing: blackgum (Cornales: Cornaceae), redbay (Laurales: Lauraceae), sugar maple (Sapindales: Aceraceae), and sweetgum (Saxifragales: Altingiaceae).

## Materials and Methods

### Genome Sizing

The procedure used to analyze nuclear DNA content in plant cells was modified from [32]. Briefly, the procedure consists of preparing suspensions of intact nuclei by chopping plant tissues in MgSO_4_ buffer mixed with DNA standards and stained with propidium iodide (PI) in a solution containing DNAase-free-RNAase. Fluorescence intensities of the stained nuclei are measured by a flow cytometer. Values for nuclear DNA content are estimated by comparing fluorescence intensities of the nuclei of the test population with those of an appropriate internal DNA standard that is included with the tissue being tested. Chicken Red blood cells (2.5 pg/2C), *Glycine max* (2.45 pg. /2C), Oryza sativa cv Nipponbare (0.96 pg/ 2C), or Arabidopsis thaliana (0.36 pg/2C) were used as the internal standard. The pellet was suspended by vortexing vigorously in 0.5 mL solution containing 10 mM MgSO_4_.7H_2_O, 50mM KCl, 5 mM HEPES, pH 8.0, 3 mM dithiothreitol, 0.1 mg / mL propidium iodide, 1.5 mg / mL DNAse-free RNAse (Roche, Indianapolis, IN) and 0.25% Triton X-100. The suspended nuclei were withdrawn using a pipettor, filtered through 30-μm nylon mesh, and incubated at 37°C for 30 min before flow cytometric analysis. Suspensions of sample nuclei were spiked with suspension of standard nuclei (prepared in above solution) and analyzed with a FACScalibur flow cytometer (Becton-Dickinson, San Jose, CA). For each measurement, the propidium iodide fluorescence area signals (FL2-A) from 1,000 nuclei were collected and analyzed by CellQuest software (Becton-Dickinson, San Jose, CA). The mean position of the G0/G1 (nuclei) peak of the sample and the internal standard were determined by CellQuest software. The mean nuclear DNA content of each plant sample, measured in picograms, was based on 1,000 scanned nuclei.

### Library preparation & Sequencing

Tissues were collected from ten species of hardwood tree. *Acer saccharum*, *Juglans nigra*, *Liquidambar styraciflua* and *Nyssa sylvatica* were collected in Boone County, Missouri from public lands not requiring sampling permission. These trees were collected from private land, with land owners permission: *Gleditsia triacanthos* in DeKalb, Tennessee; *Quercus alba* from Ava, Missouri; and *Persea borbonia* from St. Stephen, SC. Coordinates are provided in Table 1 for these trees. *Fraxinus pennsylvanica* and *F*. *americana* were provided from greenhouse stock at the Forest Service Northern Research Station. *Prunus serotina* was collected from the Bureau of Forestry at Penn Nursery, and permission was obtained.

**Table 1:** Coordinates for sampled trees.

Species	Location	°N Latitude	°W Longitude
Acer saccharum	Boone County, MO	38° 56’46.27”	92° 19’27.99”
Juglans nigra	Boone County, MO	39° 01’05.04”	92° 45’42.81”
Liquidambar styraciflua	Boone County, MO	38° 56’44.25”	92° 19’24.66”
Nyssa sylvatica	Boone County, MO	38° 57’02.75”	92° 18’33.07”
Gleditisia triacanthos	DeKalb, TN	35° 54’46.607”	85° 54’32.083”
Quercus alba	Ava, MO	36^0^57’17”	92^0^39’40”
Persea borbonia	St. Stephen, SC	33^0^21’538”	79° 58’136”

Genomic DNAs for all ten species were extracted using a modified CTAB method, and were sheared to an average fragment size of 160–300 bp, as outlined in Jennings et al. [33]. Sheared DNAs were end repaired and converted into standard Illumina sequencing libraries (Illumina, Inc., San Diego, CA) using theTruSeq v.2 Genomic DNA sequencing kit and indexed sequencing adapters. Libraries were pooled at approximately equimolar amounts, along with one additional unrelated library, and sequenced with 101 base paired-end reads on an Illumina HiSeq 2000 at Oregon State University Center for Genome Research and Biocomputing (http://www.cgrb.oregonstate.edu/). Low sequence yield and low sequence quality for green ash (Online Resource 1) required construction of a new library and a second run, on an Illumina HiSeq 2000 lane, with two other indexed libraries unrelated to this project. The combined reads from both runs are reported for green ash. All data are available through NCBI SRA [34] under project number SRP021923. Sequence statistics are available in S1 File.

### Sequence Assembly, Repeat and Gene Content Analysis

Extended fragments and non-overlapping sequences were assembled for each species with the software Abyss [35] at k-mer lengths of 19, 23, 27 and 31. Repetitive elements were identified by comparing all reconstructed read fragments for each species with RepeatMasker version 4.0.5 [36] to all known repeats from plants in the database RepBase release 19.12 [37]. Putative reads overlapping genes were identified with BLASTX [38] sequence similarity comparison of all reconstructed read fragments to a database of all plant proteins from the Swiss-Prot database [39]. Reconstructed fragments with matches with an e-value of less than 1e-5 were considered indicative of likely gene content. These reads were further compared to the transcript sequences from three model plant species: *Amborella trichopoda* version 1.0, *Arabidopsis thaliana* version TAIR10 and *Vitis vinifera* version Genoscope 145. The comparison was conducted with the program tblastx (version 2.2.26) with an e-cutotff value of 1e-5, and only the best match was kept. The transcript sequences were downloaded from Phytozome[40].

### Microsatellite discovery pipeline

Reads were trimmed of adapters, and low quality reads were removed using Trimmomatic version 0.20 with parameters to clip the TruSeq adapters (2:40:15), to quality trim with a sliding window (4:15) and to remove any sequences with less than 36 high quality bases. Further filtering was performed to remove reads where the last thirty bases of the forward and reverse read were identical. Some reads were identified where the last thirty bases of the forward and reverse read were identical; this implies overlap but in a different orientation expected from library construction protocols, possibly indicating short inserts or adapter ligation problems. These reads were removed from further consideration. Forward and reverse reads were examined for overlap in the expected orientation and if found, the original fragment was reconstructed using FLASH version 1.2.2 with parameter–t 10 [41].

Custom scripts were designed to extract SSRs from adapter-trimmed, reconstructed overlapping sequences. Repeats were reported for 2 base pair motifs occurring 8–40 times, 3 base pair motifs occurring 7–30 times and 4 base pair motifs occurring 6–20 times. Only perfect repeats were reported, and compound SSRs, i.e. two or more adjacent repeat sets with different motifs, were ignored. These constraints and an additional requirement of at least 15 bases of non-repetitive sequence on either end of the repeat region were chosen to allow for primer design. The SSR-containing reads were assembled, collapsing reads from the same locus into a single contig, using the software CAP3 with the p parameter set to 95 [42]. Sequences with compound SSRs, two or more adjacent repeat motifs, were removed. The remaining sequences were masked for low complexity regions using dustmasker level 1 [43], and primers were designed to flank the SSRS using Primer3 version 2.3.5 [44] with parameters primer_opt_size = 20, primer_min_size = 18, primer_max_size = 25, primer_num_ns_accepted = 0, primer_product_size_range = 100–200, primer_opt_tm = 60.0, primer_min_tm = 55.0, primer_max_tm = 65.0, primer_min_gc = 40, primer_max_gc = 60, primer_max_poly_x = 3, primer_gc_clamp = 2. The perl scripts are publicly available for download at https://github.com/mestato/lab_code/tree/master/hwg_gssr_scripts. The results of the analysis including primers are available in S3 File and online at http://www.hardwoodgenomics.org/content/gssrs. A flow chart of data analysis steps are provided as a visualization in S2 File.

## Results

### Genome Size

Published genome size estimates existed for only two of the ten hardwood species evaluated in this study: 489Mb for tetraploid black cherry [45], and 766Mb for white oak [46]. Red bay (*Persea borbonia)* has not been directly measured, but genome sizes were available for two other species of *Persea*, *P*. *americana* (905Mb) and *P*. *indica* (1614Mb), both diploids [47]. For redbay we used an average of these values, 1255Mb, as a rough estimate for the purpose of sequence coverage calculations.

Nuclear DNA content was estimated using flow cytometry for six of the seven remaining trees in our study, producing the following results: blackgum (1238Mb), black walnut (695Mb), honeylocust (1255Mb), green ash (975Mb), sweetgum (799Mb), and white ash (930Mb) (Table 2). The genome size of sugar maple (*Acer saccharum*) was not obtained. Although genome size estimates exist for several Acer species, there is great variability of ploidy across *Acer* [48]. Thus, we did not calculate estimated sequence coverage statistics for sugar maple.

**Table 2:** Genome size estimates obtained from flow cytometry.

Species name	2C DNA in pg (mean value)	Estimated 1n in Mbp	# genotypes; # replicates measured
*Juglans nigra* (black walnut)	1.42	695	4; 4
*Nyssa sylvatica* (blackgum)	2.53	1,238	4; 4
*Fraxinus pennsylvanica* (green ash)	1.99	975	3; 4
*Gleditsia triacanthos* (honeylocust)	2.57	1,255	2; 4
*Liquidambar styraciflua* (sweetgum)	1.63	799	6; 4
*Fraxinus americana* (white ash)	1.90	930	3; 4

Measurements are provided in picograms (pg) and millions of nucleotide base pairs (Mbp).

### Sequence yield and genome content analysis

Libraries were prepared from all ten species and run on a single Illumina lane, yielding over 210 million indexed reads. An additional yield of 64.9 million reads was obtained from a second green ash sequencing run for a combined yield of 275 million reads and 27.8 gigabases of sequence. The estimated genome coverage of the sequence data after filtering varied from a low of 0.5X in sweetgum to a maximum of 4.7X in black walnut (S1 File). Genome size estimates were obtained from the literature or through flow cytometry, described in the genome size results.

The average targeted library fragment size, 180 bases, was chosen to create overlapping forward and reverse paired end sequences, allowing the reconstruction of the sequence of the entire original fragment. The percentage of the filtered reads that could be combined to produce a single contiguous fragment ranged from a low of 4% in blackgum to a high of 93% in honeylocust. From the reconstructed fragments, four species had over 1X genome sequence coverage of the genome with a high of 2.6X in black cherry. The other six species had less than 1X coverage with a low of 4% (i.e. 0.04X) coverage of the blackgum genome (Table 3). The GC content of each genome was estimated from the reconstructed fragments, and ranged from 31.6% (honeylocust) to 38.1% (sugar maple).

**Table 3:** Statistics from low coverage whole genome sequencing and microsatellite discovery.

Species	Genome coverage (X-fold depth)	GC%	# gSSRs	# PALs	gSSRs per Mb	PALs per Mb	% of PALs matching a plant gene	% of trinucleotide PALs matching a plant gene
Black cherry	2.63	36.8	30,818	8,932	24.0	6.9	6.8%	18.0%
Black walnut	1.71	34.7	44,577	12,751	37.4	10.7	2.7%	5.6%
Blackgum	0.04	34.0	8,154	1,103	153.8	20.8	1.4%	2.5%
Green ash	0.77	34.7	13,590	2,650	18.1	3.5	2.4%	2.5%
Honeylocust	1.71	31.6	30,997	4,715	14.4	2.2	2.4%	5.9%
Redbay	1.49	37.9	56,887	18,167	30.4	9.7	1.3%	1.8%
Sugar maple	N/A	38.1	6,051	891	59.0	8.7	1.3%	1.9%
Sweetgum	0.14	36.9	7,340	1,889	63.7	16.4	1.2%	1.5%
White ash	0.34	33.9	5,325	1,079	16.8	3.4	3.2%	2.2%
White oak	0.11	33.6	6,995	1,005	84.0	12.1	1.6%	4.8%

Genome coverage and GC percentages calculated from reconstructed fragments, i.e. overlapping paired ends joined to create a single sequence. Potentially amplifiable loci (PALs) are gSSRs (genomic SSRs) that have flanking primers, allowing them to be tested for polymorphism. The rates of these markers per million bases (Mb) are calculated based on the total bases of reconstructed fragments for each species.

**Fig 1:**
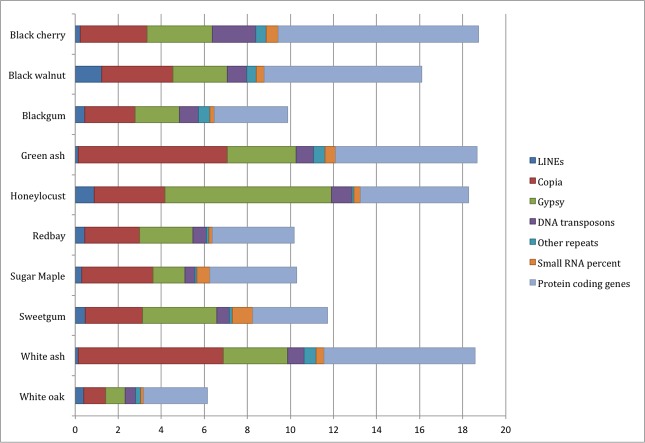
Identified repetitive elements and genes in genomic reads. The percent of reconstructed fragments with sequence similarity to known plant repetitive elements and gene sequences vary across species. The majority of identified repetitive elements originate from the retrotransposon classes of Gypsy and Copia.

The extended fragments as well as the non-overlapping read pairs were assembled at four k-mer lengths. The assemblies all spanned less than half of the estimated total genome lengths for the 10 species, with N50 lengths below 300 bases. The low percentage of the genomes covered by the assembly for each species likely resulted in repetitive DNAs collapsing into contigs within the assemblies, which could facilitate identification of classes of repeats, but not their full distribution across the genome. However, surveys utilizing the entire, unassembled low coverage read sets have frequently been utilized to survey the structure and content of genomes [11–13]. We assessed the repeat content of each genome by screening the unassembled reconstructed fragments against a database of known plant repetitive elements (Fig 1). White ash, green ash and honeylocust genomes had the highest repetitive content with 11.2%, 11.6% and 13.0% repeats, respectively. This contrasts with sugar maple, sweetgum, blackgum, red bay, and white oak genomes in which relatively low repeat content was identified, from 3.0% to 4.0% of fragments. For all 10 species, the majority of identified repeats were in the Ty1/Copia- or Gypsy-like families, with Ty1/Copia elements found more often than Gypsy elements in all species except honeylocust and sweetgum. The very large number of repeats found in honeylocust overall, 13.0%, is largely due to a significantly higher number of Gypsy elements, corresponding to 7.74% of reads.

To further characterize the genomes of these trees, the reconstructed fragments were compared to known plant protein sequences in order to find which fragments contained genes. From 3.0% (white oak) to 9.3% (black cherry) of sequences had protein matches (Fig 1). These sequences have been extracted from the genome assemblies and placed online for public access in two formats: fasta files and Excel formatted worksheets with embedded matching protein function information (http://www.hardwoodgenomics.org/content/gssrs); this data is also available in S3 File. To estimate the relative percent of each plant’s gene space that is available from the gDNA read data, the reads with homology to plant protein sequences were mapped to the gene coding sequences of three phylogenetically diverse reference species: *Arabidopsis thaliana*, *Vitis vinifera* and *Amborella trichopoda*. The percent of genes from each reference species with at least one read aligning to a protein from a hardwood tree is reported in Table 4. The percent of the gene sequences in the 10 species that aligned to the genome of *Amborella*, which is phylogenetically basal to all flowering plants, was consistently lower than to *Vitis* and *Arabidopsis*. The percent of gene sequence matches to grape, an outgroup to the rosids, and Arabidopsis, a well-annotated model rosid, were similar across all trees surveyed. Due to gene divergence and duplications across lineages, the results are only a general estimate, and all genes in model species will not be present in the tree species or vice versa. However, the results do illustrate a general pattern; at most about 40–50% of the gene sequences from reference plant genomes have homology to the tree sequenced for this project. Interestingly, even the highest level of coverage in this experiment, at 2.6X in black cherry, did not yield a larger percentage of genes for which database matches could be found. This may indicate that only about half of genes have enough sequence similarity to be mapped from the short genomic fragments generated. At the lower end of genome coverage, even lower numbers of gene matches were obtained. For blackgum, with an estimated genome coverage of .04X (or 4% of the genome), only 12–15% of model plant genes were matched by at least one gDNA read.

**Table 4:** Percent of genes represented in low coverage reconstructed fragments, based on comparison to three model plant species.

	% *Amborella* Genes Matched	% *Arabidopsis* Genes Matched	% *Grape* Genes Matched
Black Cherry	41%	52%	55%
Black Walnut	39%	49%	53%
Blackgum	12%	14%	15%
Green Ash	39%	49%	51%
Honeylocust	41%	51%	54%
Redbay	41%	49%	53%
Sugar Maple	18%	21%	23%
Sweetgum	21%	24%	27%
White Ash	32%	40%	41%
White Oak	13%	15%	17%

### gSSR identification

**Fig 2:**
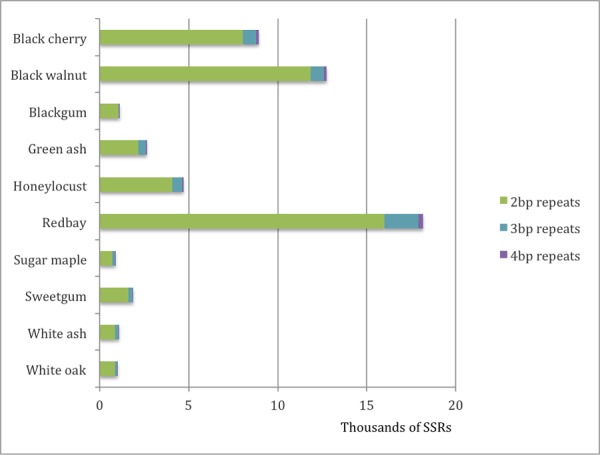
Number of PALs (potentially amplifiable loci) for each of ten hardwood tree species. Hundreds to thousands of PALs were identified for each species sequenced. For all species the most commonly identified repeat motif was 2 bases, followed by 3 base motifs. Reptitive motifs of 4 bases were found the least often.

SSRs were identified in the reconstructed fragments of genomic DNA (gSSRs) for all ten tree species sequenced. The number of gSSRs found per megabase of sequence in reconstructed gDNA fragments varied considerably between species, with nine of the ten having values ranging from 14.4 (honeylocust) to 84.0 (white oak). Blackgum was a clear outlier with 153.8 gSSRs per megabase (Table 3). For all species the dinucleotide repeats class of SSRs were far more common than trinucleotide repeats; tetranucleotide repeats were the least abundant (Fig 2). In general, motifs with all or mostly GC bases were much less abundant than AT-rich motifs, though considerable variation among species was detected (Table 5).

**Table 5:** Frequency of repeat patterns for two base pair and three base pair motif gSSRs. For both 2- and 3-base pair repeat motifs, repeats with a lower GC% was more commonly found. Shifted and reverse complement motifs were merged into a single category; e.g. AG shifted by a single base is GA, and on the reverse strand is CT or TC. Abbreviations: BC = black cherry, BW = black walnut, BG = blackgum, GA = green ash, HL = honeylocust, RB = redbay, SM = sugar maple, SG = sweetgum, WA = white ash, WO = white oak.

**SSRs with 2-base Repeat Motif**	**BC**	**BW**	**BG**	**GA**	**HL**	**RB**	**SM**	**SG**	**WA**	**WO**
AT|TA	38%	56%	74%	23%	64%	55%	51%	39%	32%	55%
AG|GA|CT|TC	57%	39%	14%	42%	22%	39%	36%	50%	49%	37%
AC|CA|TG|GT	5%	5%	12%	35%	14%	6%	13%	11%	19%	8%
GC|CG	0%	0%	0%	0%	0%	0%	0%	0%	0%	0%
**SSRs with 3-base Repeat Motif**	**BC**	**BW**	**BG**	**GA**	**HL**	**RB**	**SM**	**SG**	**WA**	**WO**
AAT|ATA|TAA|ATT|TTA|TAT	52%	59%	78%	53%	64%	37%	51%	38%	65%	58%
ATG|TGA|GAT|CAT|ATC|TCA	10%	5%	3%	8%	1%	14%	8%	4%	8%	7%
AAG|AGA|GAA|CTT|TTC|TCT	19%	26%	13%	22%	21%	32%	27%	46%	14%	23%
AGT|GTA|TAG|ACT|CTA|TAC	1%	1%	0%	3%	0%	2%	0%	0%	2%	1%
AAC|ACA|CAA|GTT|TTG|TGT	5%	2%	3%	5%	11%	6%	7%	6%	3%	9%
CCA|CAC|ACC|TGG|GTG|GGT	3%	1%	0%	6%	0%	2%	2%	2%	5%	2%
AGC|GCA|CAG|GCT|CTG|TGC	4%	2%	1%	1%	1%	2%	1%	0%	1%	0%
AGG|GAG|GGA|CCT|CTC|TCC	5%	4%	3%	2%	1%	4%	2%	3%	2%	0%
ACG|CGA|GAC|CGT|GTC|TCG	0%	0%	0%	0%	0%	0%	0%	0%	0%	0%
GGC|GCG|CGG|GCC|CCG|CGC	0%	0%	0%	0%	1%	0%	0%	0%	0%	0%

Only those SSRs for which unique, high quality primers can be designed from gDNA sequence on either side of the repeat region will be useful for genetic assays by PCR amplification and screening by fragment analysis. Despite optimization of the repeat identification parameters within our primer site identification and design program, primers could only be successfully designed for 13% of the detected gSSRs (Table 3). The species with the fewest number of PALs was sugar maple with 891 candidate gSSRs. Red bay had the highest number with 18,167.

PALS originating in gene sequences are sometimes preferred over non-genic loci, especially in genetic linkage mapping studies. Genic PALS have the advantage of tagging the functional part of the genome and may be inside of genes controlling traits of interest. However, they are less likely to be polymorphic due to high sequence conservation in coding regions [49–51]. We found a low number of PALs originating within the coding region of gene sequences; for nine of the ten species, 1% to 3% of PALS had sequence similarity to known plant genes. Black cherry had a relatively larger percentage, 6.8%, of PALs corresponding to genes. For nine of the ten species, the trinucleotide PALs were more likely to originate from genes than the 2 or 4 base pair motifs, however, for white ash a comparatively lower percentage of trinucleotide SSR primer sets originated from gene sequences (Table 3). This is a well reported phenomenon relating to the three base encoding system for amino acids; loss or gain of three bases will not shift the open reading frame [52,53].

For three of the ten tree species, redbay, sugar maple and honeylocust, amplification and polymorphism tests were conducted on a subset of the *in silico* mined PALs and resulted in identification of useful polymorphic loci that have subsequently been published [54–56]. These marker resources demonstrate that future laboratory testing of the PALs for the remaining seven species are likely to return polymorphic loci as well.

## Discussion

We report here preliminary genome characterization of ten hardwood tree species, all native to the eastern United States and previously lacking in genome sequence based resources. We illustrate the use of economical next-generation sequencing methods and flow cytometry to provide gDNA sequence for a preliminary characterization their genomes, including identification of gene sequences, and identification of microsatellite DNA markers. The coverage of sequence reads across the genome for each tree was low, but the sequences and measurements of genome size were still able to yield a glimpse of the underlying genomic structure in terms of genes and repetitive element content. The two most closely related trees, green ash and white ash, showed similar relative levels of putative repetitive elements and gene sequences (less than 0.4% divergence in any category), indicating that they may have conserved genomic structure despite the slightly diverged genome size estimates of 975Mb and 930Mb, respectively. Similarly to most sequenced model plants, Ty1/Copia and Gypsy-type retroelements dominated the repetitive DNAs identified in all of the species queried. Interestingly, the relative levels of identified repeats were only slightly correlated to total genome size as measured by flow cytometry. The inability to identify a larger percentage of reads as repetitive may be correlated to the short read length and to the divergence of repeat structures and sequences across the wide phylogenetic range spanned by the ten tree species. The random nature of shotgun sequencing across the genome may give accurate relative quantification of genes and repeats despite lower coverage. However, due to low coverage generated, the results are preliminary and less reliable for genome characterization than full genome sequencing projects; this is particularly true for five of the species surveyed that had coverage of less than 1X.

As a complementary resource and a guide for sequence coverage, cytological measurements were taken for seven species. The nuclear DNA content measurements coupled with additional knowledge of genomic repeat and gene content provide utility for rapid development of new genomic resources, for example, designing a whole genome sequencing strategy, creating and probing a clone library, or obtaining sufficient markers for mapping. The genome estimates for blackgum and sweetgum are both the first for their respective genera. The green and white ash measurements were within .05 pg of other ash estimates [48]. Honeylocust and black walnut measurements were slightly larger, 0.24 pg and 0.09 pg, respectively, than prior measurements in their genera [57,58].

The insights into repetitive elements, gene content and overall genome size provided by a shallow sequence run may be used to inform more in-depth sequencing efforts and to plan genetic mapping or association mapping studies. Many SSRs were identified in the reconstructed genome sequence fragments, further supporting the value of this approach in enabling population genetic studies with new species. Jennings et al. in 2011 reported successful identification of SSRs from multiplex DNA sequencing of seven species (2 conifer trees and 5 birds) using microsatellite-enriched libraries on a single lane of early Illumina genomic sequencing technology (the Illumina Genome Analyzer II). These authors estimated that NGS library construction and sequencing for each species cost less than $400 [21]. The work presented here takes advantage of recent sequencing technology (the Illumina HiSeq2000) that offers almost 30 times more DNA sequencing capacity, allowing the multiplex to be increased in this study to a total of ten species per lane, and increasing total genome coverage to nearly 1X, eliminating the need for microsatellite-enrichment. By eliminating the microsatellite enrichment step, we sampled across the entire genome and were able to the glean much more, unbiased sequence data for genome content analysis.

The ongoing introduction of new sequencing equipment and chemistries with significantly more data output per lane will continue to increase the number of species that can be multiplexed, while decreasing the price per species for low-coverage genome sequencing. One impediment to increasing the number of species further is variability of the depth of sequence for each species. For example, we chose to perform additional sequencing of green ash to compensate for its low representation, only 3.7%, in the original lane. However, using only the green ash data from the original sequencing lane, 3,611 gSSRs and 665 PALs were identified by the same analysis procedure. This supports the conclusion that ten species can be adequately assessed with a single Illumina lane if PALs are the only objective; additional sequencing may be required to adequately characterize genomic structure.

Beyond technical variation, the tree species vary in frequency of SSRs and repeat motif patterns. From the reconstructed reads, the PALs per megabase of raw data ranged from 0.36 to 5.67. Across all ten species, repeats were biased toward shorter, more AT-rich motifs, and repeats of only G and C bases were extremely rare. The rarity of GC repeats has been previously reported from the whole genome reference sequences of the dicot plants *Arabidopsis thaliana*, *Populus trichocarpa* and *Medicago truncatula* [59], and the Illumina platform has a known bias against sequencing GC rich reads [60].

The analysis of genome organization, the unverified *in silico* SSR set, and the genome size estimates are valuable tools to enable future genomic and genetic inquiries for forest trees. They will facilitate and complement the construction of mapping populations, genetic maps, quantitative trait loci (QTL) maps and diversity studies ongoing for many of the same tree species.

## Supporting Information

S1 File Sequence yield and estimated genome coverage prior to trimming, after trimming, and after combining reads overlapping reads. (XLSX)

S2 File A visualization of analysis steps for low coverage genome sequencing data. (TIF)

S3 File Primer pairs, predicted product size and nucleotide sequences for PALs from ten species. (XLSX)
