# Supplementary figures and images for: Preliminary Genomic Characterization of Ten Hardwood Tree Species from Multiplexed Low Coverage Whole Genome Sequencing
Source: PLoS One. 2015 Dec 23;10(12):e0145031. doi: 10.1371/journal.pone.0145031 (PMC4689444; doi:10.1371/journal.pone.0145031)

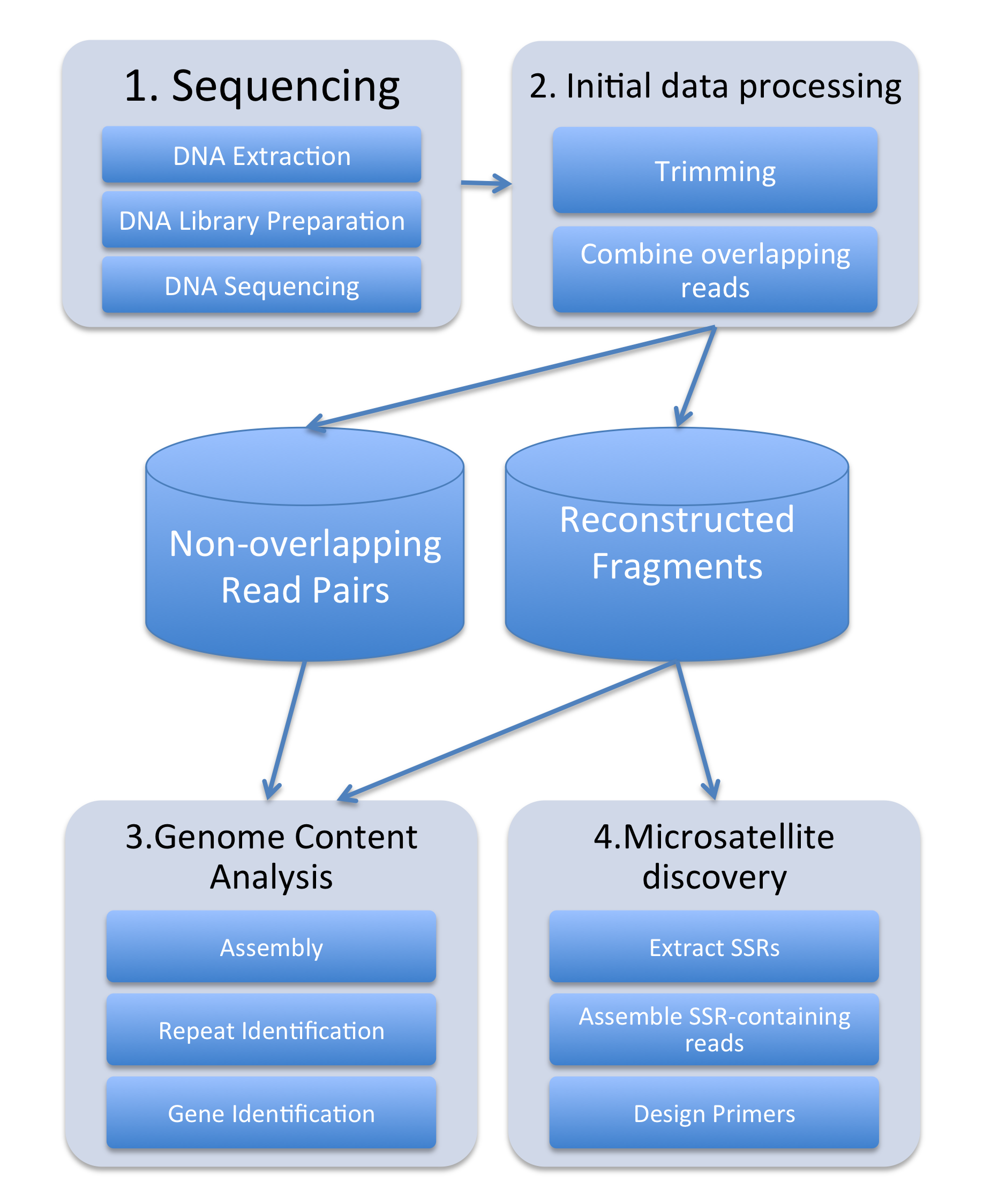

Supplement: S2 File — (TIF) [file pone.0145031.s002.tif]
